# The Root Extract of *Pueraria lobata* and Its Main Compound, Puerarin, Prevent Obesity by Increasing the Energy Metabolism in Skeletal Muscle

**DOI:** 10.3390/nu9010033

**Published:** 2017-01-04

**Authors:** Hyo Won Jung, An Na Kang, Seok Yong Kang, Yong-Ki Park, Mi Young Song

**Affiliations:** 1 Department of Herbology, College of Korean medicine, Dongguk University, Dongdaero 123, Gyeongju-si 38066, Korea; tenzing2@hanmail.net (H.W.J.); ank4200@gmail.com (A.N.K.); seokppo2@hanmail.net (S.Y.K.); yongki@dongguk.ac.kr (Y.-K.P.); 2 Korean Medicine R&D Center, College of Korean medicine, Dongguk University, Dongdaero 123, Gyeongju-si 38066, Korea; 3 Department of Rehabilitation Medicine of Korean Medicine, College of Korean Medicine, Dongguk University, Dongdaero 123, Gyeongju-si 38066, Korea

**Keywords:** Radix *Pueraria lobata*, obesity, puerarin, skeletal muscle, C2C12, PGC-1α, AMPK

## Abstract

Radix *Pueraria lobata* (RP) has been reported to prevent obesity and improve glucose metabolism; however, the mechanism responsible for these effects has not been elucidated. The mechanism underlying anti-obesity effect of RP was investigated in high-fat diet (HFD) induced obese mice and skeletal muscle cells (C2C12). Five-week-old C5BL/6 mice were fed a HFD containing or not containing RP (100 or 300 mg/kg) or metformin (250 mg/kg) for 16 weeks. RP reduced body weight gain, lipid accumulation in liver, and adipocyte and blood lipid levels. In addition, RP dose-dependently improved hyperglycemia, insulinemia, and glucose tolerance, and prevented the skeletal muscle atrophy induced by HFD. Furthermore, RP increased the peroxisome proliferator-activated receptor gamma coactivator-1 alpha (PGC-1α) expression and phosphorylation of adenosine monophosphate-activated protein kinase (AMPK) in skeletal muscle tissues. RP and its main component, puerarin, increased mitochondrial biogenesis and myotube hypertrophy in C2C12 cells. The present study demonstrates that RP can prevent diet-induced obesity, glucose tolerance, and skeletal muscle atrophy in mouse models of obesity. The mechanism responsible for the effect of RP appears to be related to the upregulation of energy metabolism in skeletal muscle, which at the molecular level may be associated with PGC-1α and AMPK activation.

## 1. Introduction

The current obesity epidemic is one of the greatest public health concerns of our century [1]. It has been estimated that the number of obese individuals could increase to 1.12 billion in 2030, which would account for 20% of the world’s adult population [2]. Obesity has been linked with numerous health-related pathologies, especially metabolic syndrome. Obesity and metabolic syndrome are known to be caused by a lack of energy homeostasis [3]. All anti-obesity drugs currently approved by the US FDA work by reducing energy intake. In fact, no approved drug targets energy expenditure, that is, modulates cellular bioenergetics [4].

Recently, many studies have been focusing on skeletal muscle as a means of treating obesity and metabolic syndrome for several reasons. First, energy expenditure takes place largely within mitochondria, which are abundant in skeletal muscle. In fact, the oxidative capacity of skeletal muscle is predominantly dependent on mitochondria [4]. Second, skeletal muscle is known to be important in the contexts of insulin resistance and glucose metabolism. Insulin resistance is associated with myocellular lipid accumulation, and it has been known insulin resistance to be caused by impaired oxidative capacity in skeletal muscle [5,6]. Third, recent studies have shown obesity to be associated with skeletal muscle loss, which is also referred to as a sarcopenic obesity [7]. Furthermore, skeletal muscle loss is strongly associated with obesity-induced insulin resistance in both young and old adults, which underscores the important role of low muscle mass as an independent risk factor of metabolic disease [8].

In skeletal muscle, peroxisome proliferator-activated receptor gamma coactivator-1α (PGC-1α) plays a central regulatory role in cellular energy metabolism, that is, it upregulates oxidative metabolism and stimulates mitochondrial biogenesis [9]. Recently, it was reported PGC-1α is an important mediator of muscle mass and that it protects against skeletal muscle atrophy [10]. Adenosin monophosphate-activated protein kinase (AMPK) is another enzyme central to cellular energy metabolism. AMPK detects the nutrient status of cells and helps regulate glucose transport, fatty-acid oxidation, and metabolic adaptations in skeletal muscle [11]. Furthermore, AMPK has been reported to affect PGC-1α activity directly by phosphorylation [12].

Radix *Pueraria lobata* (RP) is the dried root of *Pueraria lobate* (Willd.) Ohwi, which is used traditionally to treat diarrhea, muscle stiffness, thirst, and diabetes in East Asia [13], and recently was made commercially available as a western dietary supplement. RP is a rich source of isoflavone glucosides and puerarin is the most abundant constituent of RP [14]. Previous studies have reported that chronic administration of RP extract improved glucose tolerance and decreased fasting plasma glucose levels in ob/ob mice [15], and that puerarin supplementation reduced body weight gain and lipid levels in liver and serum of high-fat-diet (HFD) fed-induced obese mice [16]. However, the mechanisms responsible for anti-obesity of RP extract or its beneficial effect on glucose metabolism have not been determined. Interestingly, in a previous study, we found that RP extract increased glucose uptake and ATP levels in mouse skeletal muscle cells [17] and based on this evidence, we hypothesized that RP extract might prevent obesity by regulating energy metabolism in skeletal muscle.

Therefore, the present study was performed to investigate the mechanism responsible for the anti-obesity effects of RP extract in HFD-induced obese mice and in C2C12 mouse skeletal muscle cells.

## 2. Experimental Section

### 2.1. Preparations

RP was purchased from Kwangmyungdang Medicinal Herbs (Ulsan, Korea), and authenticated by Professor Y.-K. Park, a medical botanist at the Department of Herbology, College of Korean Medicine, Dongguk University, Republic of Korea. RP extract was prepared using a standard procedure. In brief, dried RP (200 g) was ground, boiled in purified drinking water for 3 h, filtered through a two layers of Whatman No. 3 filter paper, and concentrated under vacuum (yield 32.0%). The dried powder obtained (RP extract) was stored at −80 °C, and dissolved in distilled water prior to assays.

### 2.2. HPLC Analysis

HPLC was performed using an Agilent 1220 Infinity LC System (Agilent Technologies, Santa Clara, CA, USA) and an Agilent Eclipse XDB-C18 column (4.6 mm × 250 mm, 5 μm) (Agilent Technologies). HPLC quality water and methanol were used as a mobile phase with gradient elution at a flow rate of 0.5 mL/min. 20 μL of RP extract (500 μg/mL) or puerarin (10 μM) (the standard compound, Sigma-Aldrich, St. Louis, MO, USA) were injected and detected at 250 nm using a DAD detector.

### 2.3. Animals and Experimental Design

C5BL/6 mice (aged five weeks, male) were purchased from Samtako Bio Korea (Geonggido, Korea) and kept in individual cages at a temperature of 22–23 °C under a 12 h-light/12 h-dark cycle. All experimental procedures were approved beforehand by the Committee on the Ethics of Animal Experiments of Dongguk University (Permit number: 2016-0624).

Mice were randomly divided into five groups of five animals after being allowed seven days of adaptation, as follows; (1) a normal diet group (the ND group; 18 kcal% fat, 2018S Envigo, AH, UK); (2) a high fat diet group (the HFD group; 60 kcal% fat, D12492, Research Diets, New Brunswick, NJ, USA); (3) a HFD plus RP 100 mg/kg/day administration group (the RP 100 group); (4) a HFD plus RP 300 mg/kg/day administration group (the RP 300 group); and (5) a HFD plus metformin 250 mg/kg/day administration group (the Met group). The RP or Met group was administered orally by gavage, the ND or HFD group was given an equal volume of distilled water by gavage. Body weight, food consumption, blood glucose levels, and rectal temperatures were measured weekly. An energy efficiency ratio was calculated as the weight gain (g) during the experimental period divided by the cumulative energy intake over the same period (kcal). After 16 weeks of drug administration, mice were fasted overnight and sacrificed. Blood samples were collected by cardiac puncture, and serum was obtained by centrifuging at 5000 *g* for 15 min after sacrifice. Livers, white adipose tissues (epididymal fat), pancreases, and gastrocnemius muscle tissues were collected and weighed.

### 2.4. Glucose Tolerance Test

Oral glucose tolerance testing (OGTT, 2 g/body weight, kg) was carried out after the 16-week feeding and gavage period. All mice were fasted overnight before OGTT. Blood samples were taken before and 0, 15, 30, 60, 90, and 120 min after glucose administration. Blood glucose levels were determined by Accu-Check kit (Roche Diagnostics, Basel, Switzerland).

### 2.5. Serum Analysis

Serum insulin levels were analyzed using a mouse insulin ELISA kit (EMD Millipore Corporation, MA, USA) and total cholesterol (TC), high-density lipoprotein (HDL) cholesterol, and triglycerides (TG) were determined using enzymatic methods available as commercial kits (Asan Pharm. Co., Seoul, Korea). Aspartate aminotransferase (AST) and alanine aminotransferase (ALT) were also analyzed using appropriate commercial kits (Asan Pharm. Co., Seoul, Korea).

### 2.6. Histological Analysis

Liver, epididymal white adipose tissue, and skeletal muscle tissue were fixed in 10% formalin, stained with hematoxylin and eosin (H&E), and visualized under a Leica DM 2500 microscope (Leica, Wetzlar, Germany). The cross sectional area of adipose tissue and skeletal muscle were quantified using ImageJ analysis software [18].

### 2.7. Lipid Accumulation Analysis in Liver

Oil droplet content of liver tissue was determined by oil red O staining. Optimal cutting temperature-embedded liver tissue was cut into 10 μm sections, which were then mounted on cryostat clear slides. Tissues were allowed to dry for 1 h, hydrated in distilled water for 5 min, dipped in absolute propylene glycol solution for 2 min, stained with oil red O working solution (Sigma, Ronkonkoma, NY, USA) for 1 h, dipped in 85% propylene glycol solution for 1 min, and rinsed with distilled water. Sections were visualized under a Leica DM 2500 microscope.

### 2.8. C2C12 Cell Cultures and Treatment

C2C12 cells (CRL-1772, ATCC, Manassas, VA, USA) were cultured in DMEM (Invitrogen, Grand Island, NY, USA) supplemented with 10% FBS (Invitrogen) and a penicillin/streptomycin mix (Invitrogen). For differentiation, C2C12 myoblasts were cultured till confluent and then were incubated with the media containing was 2% horse serum (Invitrogen). Cells were cultured in this medium for four days with medium replacement every 24 h. Differentiated C2C12 myotubes were treated with or without RP (0.2 or 0.5 mg/mL), puerarin (10 or 20 µM), or metformin (2.5 mM) for 24 h. The concentration of RP or puerarin for treatment in C2C12 myotubes was determined by MTT assay, and used the non-toxic range.

### 2.9. Western Blot

Protein samples from skeletal muscle tissue or C2C12 cells were obtained by lysis in ice-cold lysis buffer containing 150 mM NaCl, 50 mM Tris-HCl, 1 mM EDTA, 50 mM NaF, 10 mM Na4P2O7, 1% IGEPAL, 2 mM Na_3_VO_4_, 0.25% protease inhibitor cocktail, and 1% phosphatase inhibitor cocktail (Sigma-Aldrich, St. Louis, MO, USA). Homogenates were centrifuged at 12,000 *g* for 20 min at 4 °C. For western blotting, 50 and 10 µg of protein samples from or cell cultures were used, respectively. Antibodies and their sources were as follows; anti-phospho-AMPKα (Thr 172), anti-AMPKα, anti-acetyl-CoA carboxylase (ACC) (Cell Signaling Technology, Danvers, MA, USA), anti-PGC1α, anti-mitochondrial transcription factor (TFAM), anti-nuclear respiratory factor-1 (NRF-1) and myosin heavy chain (MyHC) (Santa Cruz Biotechnology, Santa Cruz, CA, USA), and anti-β-actin (Sigma-Aldrich). Western detection reagent (GE Healthcare Bio-Sciences, Pittsburgh, PA, USA) was used to develop the bands, which were quantified by densitometry using Image J.

### 2.10. Immunofluorescence Analysis

C2C12 cells were fixed in 4% formaldehyde in PBS, permeabilized with 0.5% Triton X-100, and blocked with PBS containing 5% bovine serum albumin. After three washes with PBS, cells were incubated overnight at 4 °C with an anti-MyHC antibody (Santa Cruz Biotechnology, Santa Cruz, CA, USA), and then washed with PBS at room temperature. Cells were incubated with Alexa Fluor 488 goat anti-rabbit IgG (Life technologies, Carlsbad, CA, USA) for 1 h at room temperature, and washed with PBS. Cells were mounted on a coverslip with a drop of mounting medium containing DAPI (Vector laboratories, Burlingame, CA, USA). Cells were observed using fluorescence Leica DM 2500 microscope (Leica, Wetzlar, Hessen, Germany)

### 2.11. ATP Contents

Total ATP contents were determined using the ATP calorimetric assay kit (BioVision, Inc., Milptas, CA, USA). ATP concentrations were calculated using the manufacturer’s protocol, the absorbance was measured at 570 nm.

### 2.12. Statistical Analysis

The analysis was conducted using two-way (for OGTT results) or one-way ANOVA followed by Tukey’s post hoc test in GraphPad Prism program ver 5.0 (GraphPad Software, La Jolla, CA, USA). Results are presented as means ± standard errors of the mean (SEM). Statistical significance was accepted for *p* values < 0.05.

## 3. Results

### 3.1. HPLC Analysis of RP Extract

**Figure 1:**
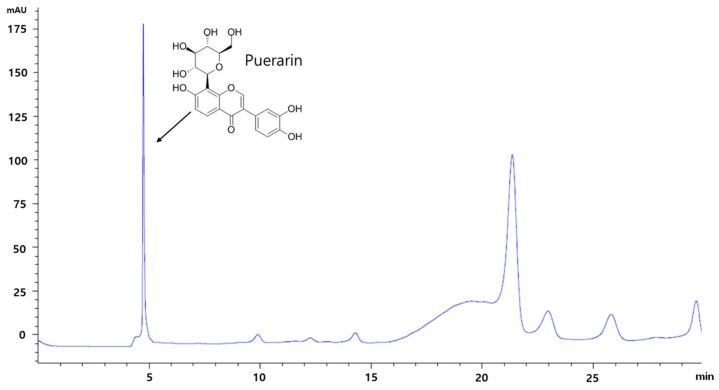
The pattern of puerarin in RP extract by HPLC analysis. RP, Radix *Pueraria lobata*; HPLC, High Performance Liquid Chromatography.

HPLC analysis revealed that RP extract contained puerarin which is known to be the main component of RP (Figure 1).

### 3.2. RP Extract Reduced Body Weight Gain

**Figure 2:**
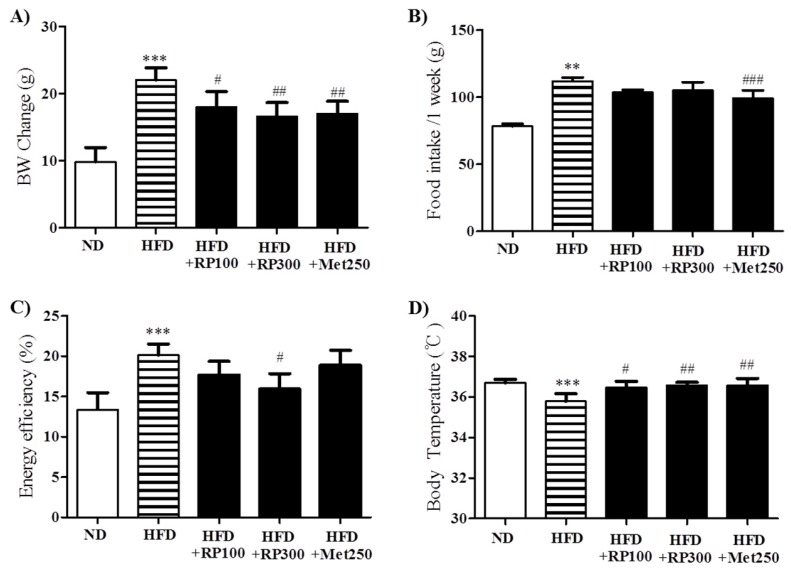
Effects of RP extract on body weight. (**A**) Body weight; (**B**) Food intake; (**C**) Energy efficiency rate; and (**D**) Body temperature were measured in C5BL/6 mice during the 16-week study period. Results are presented as means ± standard errors of the mean (SEM) (*n* = 5). *** *p* < 0.001 and ** *p* < 0.01 versus the normal chow fed controls (ND group); ^###^ *p* < 0.001, ^##^ *p* < 0.01, ^#^ *p* < 0.05 vs. the HFD mice. RP, Radix *Pueraria lobata*; ND, normal diet; HFD, high fat diet; BW, body weight.

After the 16-week feeding period, HFD mice showed significant increases in body weight as compared with ND mice. RP extracts at 100, and 300 mg/kg and metformin at 250 mg/kg significantly reduced this body weight gain (Figure 2A). RP extract treatment did not affect food intake, while metformin significantly reduced food intake compared to HFD control group (Figure 2B), energy efficiency was lower in RP extract (300 mg/kg) treatment group than in the HFD group (Figure 2C). Furthermore, HFD mice were significantly hypothermic as compared with ND mice, and RP extract significantly increased body temperatures by the area under the curve (AUC) (Figure 2D). In addition, RP supplementation significantly inhibited liver, pancreas, and epididymal adipose tissue weight increases (Table 1).

### 3.3. RP Extract Reduced Obesity-Induced Lipid Accumulation

**Figure 3:**
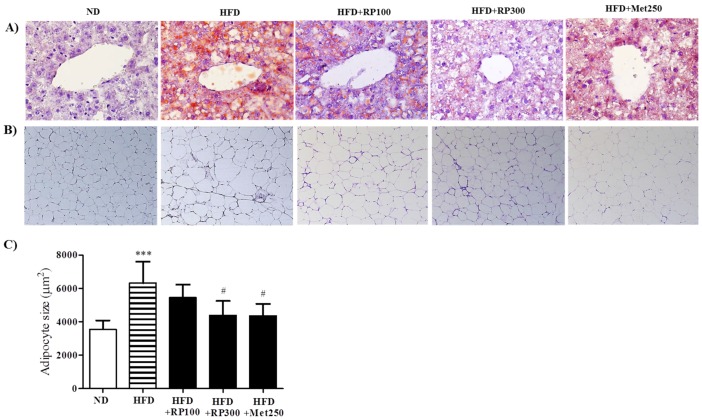
Effects of RP extract on lipid accumulations in livers and adipose tissues. (**A**) Oil red O stained liver tissues; (**B**) H&E stained epididymal adipose tissues, original magnification 200×; (**C**) Adipocyte sizes in epididymal adipose tissues. Results are presented as means ± standard errors of the mean (SEM) (*n* = 5) *** *p* < 0.001 vs. the normal chow fed controls (ND group); ^#^ *p* < 0.05 vs. the HFD mice. RP, Radix *Pueraria lobata*; ND, normal diet; HFD, high fat diet.

RP extract dose-dependently decreased lipid accumulation in livers as determined by oil-red-O staining (Figure 3A). In adipose tissues, adipocyte areas were significantly smaller in the RP extract (300 mg/kg) treatment group than in the HFD group (Figure 3B,C). Total cholesterol HDL-cholesterol, AST and ALT levels were significantly aggravated by HFD feeding, and RP extracts (100 and 300 mg/kg) treatment groups had significantly improved serum levels of both. However, triglyceride levels were unaffected by the HFD (Table 1).

### 3.4. RP Extract Improved Obesity-Induced Glucose Tolerance

**Figure 4:**
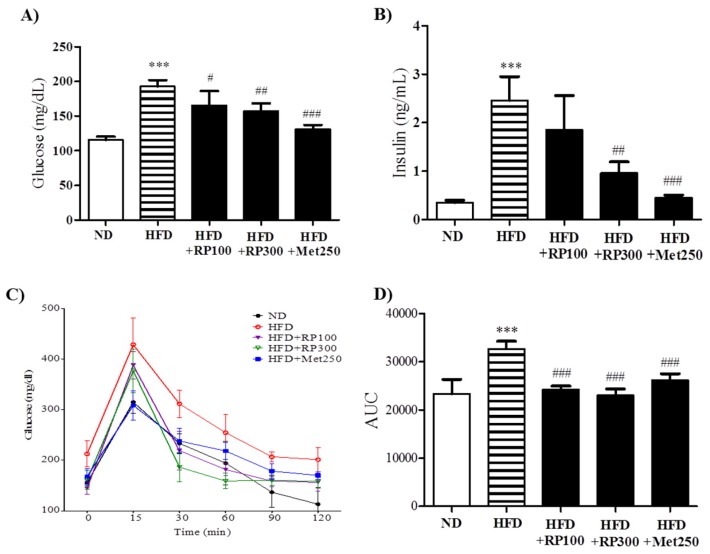
Effects of RP extract on glucose tolerance and insulinemia. (**A**) Fasting serum glucose; (**B**) Serum insulin; (**C**) Oral glucose tolerance test (OGTT); (**D**) Area under the curve in OGTT. Results are presented as means ± standard errors of the mean (SEM) (*n* = 5). *** *p* < 0.001 vs. the normal chow fed controls (ND group); ^###^ *p* < 0.001, ^##^ *p* < 0.01, ^#^ *p* < 0.05 vs. the HFD mice. RP, Radix *Pueraria lobata*; ND, normal diet; HFD, high fat diet; AUC, area under the curve.

Hyperglycemia, hyperinsulinemia, and glucose tolerance are associated with obesity and metabolic syndrome [3]. RP extract administration significantly and dose-dependently reduced fasted plasma glucose and insulin levels (Figure 4A,B). OGTT was used to examine the effect of RP on glucose tolerance. HFD mice displayed significant impairment of glucose tolerance from 0 to 120 min after glucose administration, whereas mice in RP extracts (100 and 300 mg/kg) treatment groups showed significantly better glucose tolerance as determined by the area under the curve (AUC) analysis (Figure 4C,D).

### 3.5. RP Extract Protected against Obesity-Induced Skeletal Muscle Atrophy and Improved Energy Metabolism

**Figure 5:**
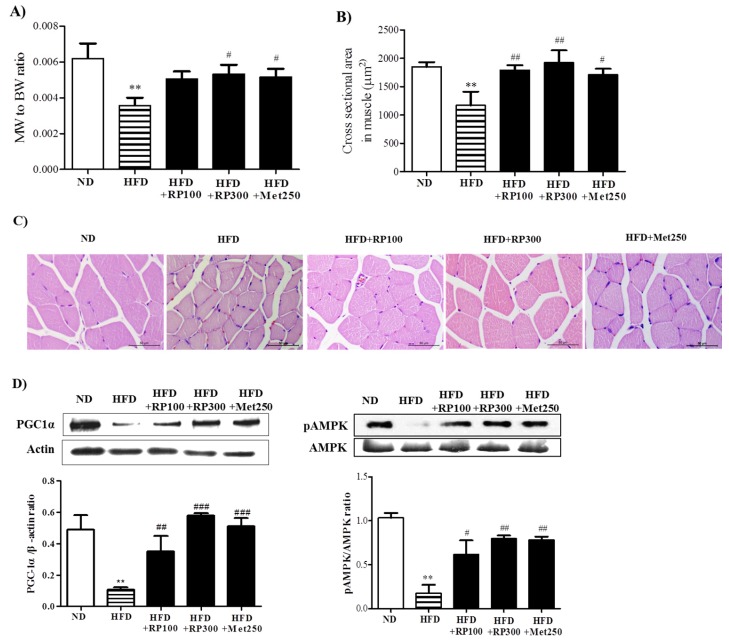
Effect of RP extract on skeletal muscle atrophy and energy metabolism. (**A**) Muscle (gastrocnemius) to body weight ratios; (**B**) Cross sectional areas of gastrocnemius muscle fibers; (**C**) Muscle fiber morphologies, original magnification 400×; (**D**) PGC-1α and pAMPK protein levels in skeletal muscle tissues. Results are presented as means ± standard errors of the mean (SEM) (*n* = 5). ** *p* < 0.01 vs. the normal chow fed controls (ND group); ^###^ *p* < 0.001, ^##^ *p* < 0.01 and ^#^ *p* < 0.05 vs. the HFD mice. RP, Radix *Pueraria lobata*; BW, body weight; MW, muscle weight; ND, normal diet; HFD, high fat diet; PGC-1α, peroxisome proliferator-activated receptor gamma coactivator-1 alpha; AMPK, adenosine monophosphate-activated protein kinase.

Skeletal muscle atrophy is characterized by reduction in muscle mass and fiber size. We found the ratio of skeletal muscle weight to whole body weight was lower in the HFD group than in the ND group, and that this reduction was ameliorated in the RP extract (300 mg/kg) treatment group (Figure 5A). Similarly, histological examinations of gastrocnemius cross sections showed that mean muscle fiber diameter was greater in the RP groups than in the HFD group (Figure 5B,C).

It has been reported PGC-1α has a significant beneficial effect on whole-body metabolism [9], and that it prevents muscle wasting [10]. To understand the cellular basis of ameliorated lipid accumulation and inhibited atrophy in RP extract, we assessed the activation of PGC1α in skeletal muscle. AMPK activities were also determined by examining phosphorylation status, because it has been reported AMPK activation elevates PGC-1α protein levels [12], and because PGC-1α and AMPK are key players in the regulation of energy metabolism at the cellular and whole-body levels [19]. As illustrated in Figure 5D, RP extracts dose-dependently increased the levels of PGC-1α and phosphorylated AMPK protein, whereas both were downregulated by HFD.

### 3.6. RP Extract and Puerarin Improved Mitochondrial Biogenesis and Myotube Hypertrophy in C2C12 Skeletal Muscle Cells

**Figure 6:**
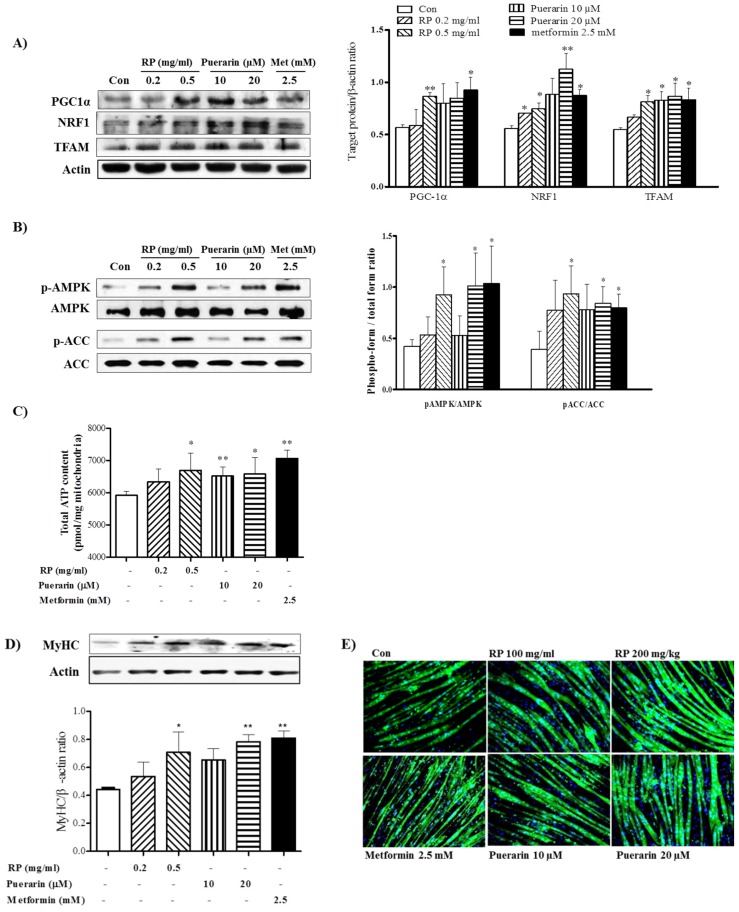
Effects of RP extract and puerarin on mitochondrial biogenesis and myotube hypertrophy in C2C12 myotubes. C2C12 myotubes were treated without or with without RP 0.2 mg/mL, RP 0.5 mg/mL, 10 µM puerarin, 20 µM puerarin, or 2.5 mM metformin. (**A**) PGC-1α, NRF1, TFAM, acitin; (**B**) AMPK and ACC protein levels; (**C**) total ATP; (**D**) Myosin heavy chain (MyHC) protein levels and (**E**) Myotube morphology with immunofluorescence analysis. Results are presented as means ± standard errors of the mean (SEM) of three independent experiments. ** *p* < 0.01 and * *p* < 0.05 vs. non-treated controls. RP, Radix *Pueraria lobata*; PGC-1α, peroxisome proliferator-activated receptor gamma coactivator-1 alpha; NRF-1, nuclear respiratory factor-1; TFAM, mitochondrial transcription factor; AMPK, adenosine monophosphate-activated protein kinase; ACC, acetyl-CoA carboxylase; ATP, adenosine triphosphate.

To assess the mechanism responsible for the anti-obesity effects of RP extract in mice, we investigated its regulatory effects on mitochondrial biogenesis and myotube hypertrophy in C2C12 cells. Accordingly, we investigated the activities of PGC-1α, AMPK, and mitochondrial biogenesis in C2C12 myotubes treated with RP extract or puerarin. In C2C12 cells, treatment with RP or puerarin increased levels of PGC-1α and the levels of NRF-1 and TFAM, the transcription factors involved in mitochondrial biogenesis (Figure 6A), and similarly induced the activation of AMPK and ACC (Figure 6B). Furthermore, RP extract and puerarin dose-dependently increased ATP levels (Figure 6C). These results indicate that RP extract and puerarin can increase mitochondrial biogenesis and energy metabolism by activation of the PGC-1α and AMPK in skeletal muscle cells.

Next, we investigated regulatory effects on myotube hypertrophy which is associated with protecting skeletal muscle atrophy. RP extract (0.5 mg/mL) and puerarin (20 μM) increased the protein levels of MyHC in C2C12 myotubes (Figure 6D). Furthermore, in line with this increase, immunofluorescence analysis showed hypertrophic morphological changes in MyHC-positive newly formed myotubes after RP or puerarin treatment (Figure 6E).

## 4. Discussion

The present study was undertaken to investigate the mechanism underlying the anti-obesity effects of RP extract in high-fat diet (HFD) induced obese mice and on skeletal muscle cells (C2C12). We found that RP extract improved diet-induced obesity, glucose tolerance, insulin resistance, and skeletal muscle atrophy, and that these effects were probably related to the upregulation of energy metabolism in skeletal muscle induced by the increased expression of PGC-1α.

Considerations of skeletal muscle are crucial in the contexts of energy expenditure [20] and insulin sensitivity [5,6]. In fact, muscle reportedly accounts for 20%–30% of total resting oxygen uptake [20] and for up to 80% of post-prandial insulin-stimulated glucose disposal in healthy individuals [21]. Furthermore, a 10% increase in skeletal muscle mass to total body weight ratio has been reported to be associated with a 11% reduction in the risk of insulin resistance [22]. Therefore, both increase of oxidative capacity and prevention of skeletal muscle atrophy are viewed as being crucial in terms of the prevention of obesity and metabolic syndrome. Moreover, previous studies have shown that RP extract and its main component, puerarin reduced obesity and insulin resistance [15,16]. However, the detailed mechanism of action involved has not been previously addressed with scientific evidence.

In the present study, mice fed a HFD were used as a model of obesity and glucose tolerance. After 16 weeks of HFD feeding, a successful animal model was established, as has been previously described [23,24]. We used the doses of RP extract at 100 or 300 mg/kg based on a previous study [25]. RP dose-dependently prevented HFD-induced body weight increases despite no significant change in food intake. It was observed that RP treated mice had a higher body temperature and lower energy efficiency rate than HFD mice. In a previous study, body temperature was observed to be positively correlated with energy expenditure and oxygen consumption [26,27]. Although, we did not measure these parameters in the present study, we would argue that the observed anti-obesity effect of RP accompanying an increase in body temperature and decrease in energy efficiency rate is commensurate with an increase in energy metabolism. On the other hand, metformin (the positive control) induced body weight loss with reduction of food intake, which was expected as metformin has been shown to reduce weight gain and to have an anorexic effect, probably due to the central regulation of appetite [28,29,30,31]

In addition, hyperglycemia, insulinemia, and glucose tolerance were dose-dependently improved by RP extract administration. In a previous study, this extract was found to reduce glucose tolerance in C57BL/6 mice, but no positive control was included [15]. Accordingly, in the present study metformin was included as a positive control, and we found no significant difference between the effect of metformin and that of RP extract on glucose tolerance test using OGTT.

As mentioned above, skeletal muscle mass importantly contributes to energy expenditure [20] and glucose metabolism [5]. Previous studies have shown a HFD causes significant skeletal muscle atrophy [24,32]. In the present study, RP extract supplementation prevented skeletal muscle atrophy induced by HFD. In addition, our in vitro assay confirmed the significant effect of RP on protection of skeletal muscle atrophy. MyHC is the major structural protein in myotubes, therefore it is an important differentiation maker in neo-formed myotubes [33]. Our immunoblot and immunochemistry assays demonstrated that treatment with RP extract or puerarin increased MyHC expression and myotube hypertrophy.

Our findings indicate that RP extract is a novel inducer of PGC-1α in vivo and in vitro. HFD-induced obese mice exhibited diminished levels of PGC-1α, whereas RP extract-treated mice exhibited increased PGC-1α expression in skeletal muscle, RP extract, and puerarin increased PGC-1α expression in C2C12 myotubes. Furthermore, treatment with RP increased the levels of AMPK phosphorylation. Therefore, our data suggest that RP treatment increased AMPK activation leading to downstream increase in PGC-1α protein levels. PGC-1α and AMPK play critical roles in the maintenance of glucose, lipid, and energy homeostasis and are likely to be involved in the pathogenic conditions, such as, obesity [19]. Metformin is known to increase PGC-1α protein levels and oxidative enzyme activities via AMPK phosphorylation in skeletal muscle [34]. Our findings demonstrate that RP extract treatment significantly and dose-dependently increased PGC-1α protein levels and AMPK phosphorylation in skeletal muscle tissues and cells. PGC-1α and AMPK is a major regulator of mitochondrial biogenesis [35], we observed RP and puerarin also increased mitochondrial biogenesis in C2C12 cells.

Recently, it was reported that PGC-1α importantly mediates muscle mass [10] and increases oxidative (type 1) fiber differentiation [36]. Transcript levels of PGC-1α have been reported to be markedly lower in mouse models of muscle atrophy induced by obesity, diabetes, or denervation [24,37,38], whereas energy restriction or leptin administration enhances skeletal muscle PGC-1α expression in rodents and humans [38,39]. Furthermore, PGC-1α is closely associated with myogenic differentiation in C2C12 cells [40]. Therefore, we hypothesize that activation of PGC-1α by RP may inhibit muscle atrophy in vivo and stimulate myotube formation in vitro.

Taken together, we found that RP extract ameliorated lipid accumulation and prevented skeletal muscle loss, which suggests RP acted to coordinate catabolic and anabolic metabolism in the skeletal muscles in obese mice. However, the mechanism responsible for the RP extract-induced expressional modulation of PGC-1α and AMPK induced by RP treatment was not fully investigated, and remains a topic for future study.

## 5. Conclusions

In conclusion, the present study demonstrates that RP extract can prevent diet-induced obesity, glucose tolerance, and skeletal muscle atrophy in a mouse model of obesity. In addition, RP extract and its main component, puerarin, greatly improved mitochondrial function and myotube differentiation in skeletal muscle cells in vitro. The mechanism responsible for these effects of RP action is considered to be related to the promotion of energy metabolism in skeletal muscle probably in association with increased PGC-1α levels. It would appear that RP has potential application for reasons of the prevention and treatment of obesity and metabolic syndrome.

## Figures and Tables

**Table 1:** Effect of RP extract on tissue weight and serum profiles.

	ND	HFD	HFD + RP 100	HFD + RP 300	HFD + Met 250
**Tissue weight (g)**					
Liver	1.50 ± 0.06	2.24 ± 0.35 ***	2.14 ± 0.17	1.57 ± 0.22 ^##^	1.46 ± 0.17 ^###^
Pancreas	0.33 ± 0.05	0.51 ± 0.03 ***	0.34 ± 0.02 ^###^	0.34 ± 0.03 ^###^	0.35 ± 0.05 ^###^
Epididymal fat	0.59 ± 0.05	1.40 ± 0.15 ^***^	1.12 ± 0.15 ^#^	1.00 ± 0.15 ^##^	0.92 ± 0.09 ^###^
**Serum**					
ALT (IU/L)	39.1 ± 5.24	81.0 ± 9.40 ***	54.6 ± 10.43 ^###^	32.80 ± 5.48 ^###^	49.9 ± 3.50 ^###^
AST (IU/L)	50.7 ± 2.87	81.8 ± 5.94 ***	66.90 ± 5.76 ^##^	46.10 ± 7.72 ^###^	59.3 ± 2.11 ^###^
TG (mg/dL)	250.10 ± 3.11	248.66 ± 3.08	245.89 ± 6.93	240.83 ± 2.92	243.19 ± 1.82
TC (mg/dL)	205.27 ± 14.58	242.67 ± 12.94 **	229.33 ± 10.97	196.33 ± 22.03 ^##^	218.33 ± 13.79
HDL-C (mg/dL)	72.81 ± 8.72	43.90 ± 12.87 **	65.54 ± 10.98 ^#^	106.51 ± 10.62 ^###^	67.77 ± 4.20 ^#^

Results are presented as means ± standard errors of the mean (SEM) (*n* = 5). *** *p* < 0.001, ** *p* < 0.01 versus normal chow fed controls (the ND group); ^###^ *p* < 0.001, ^##^ *p* < 0.01, ^#^ *p* < 0.05 versus the high fat diet fed mice (the HFD group). RP, Radix *Pueraria lobata*; ALT, alanine aminotransferase; AST, aspartate aminotransferase; TG, triglycerides; TC, total cholesterol; and HDL-C, high-density lipoprotein cholesterol.
